# Outcome disparities in African American women with triple negative breast cancer: a comparison of epidemiological and molecular factors between African American and Caucasian women with triple negative breast cancer

**DOI:** 10.1186/1471-2407-14-62

**Published:** 2014-02-04

**Authors:** Lori A Sturtz, Jen Melley, Kim Mamula, Craig D Shriver, Rachel E Ellsworth

**Affiliations:** 1 Clinical Breast Care Project, Windber Research Institute, Windber, PA, USA; 2 Clinical Breast Care Project, Walter Reed National Military Medical Center, Bethesda, MD, USA; 3 Clinical Breast Care Project, Henry M. Jackson Foundation for the Advancement of Military Medicine, Windber, PA, USA

## Abstract

**Background:**

Although diagnosed less often, breast cancer in African American women (AAW) displays different characteristics compared to breast cancer in Caucasian women (CW), including earlier onset, less favorable clinical outcome, and an aggressive tumor phenotype. These disparities may be attributed to differences in socioeconomic factors such as access to health care, lifestyle, including increased frequency of obesity in AAW, and tumor biology, especially the higher frequency of triple negative breast cancer (TNBC) in young AAW. Improved understanding of the etiology and molecular characteristics of TNBC in AAW is critical to determining whether and how TNBC contributes to survival disparities in AAW.

**Methods:**

Demographic, pathological and survival data from AAW (n = 62) and CW (n = 98) with TNBC were analyzed using chi-square analysis, Student’s t-tests, and log-rank tests. Frozen tumor specimens were available from 57 of the TNBC patients (n = 23 AAW; n = 34 CW); RNA was isolated after laser microdissection of tumor cells and was hybridized to HG U133A 2.0 microarrays. Data were analyzed using ANOVA with FDR <0.05, >2-fold difference defining significance.

**Results:**

The frequency of TNBC compared to all BC was significantly higher in AAW (28%) compared to CW (12%), however, significant survival and pathological differences were not detected between populations. Gene expression analysis revealed the tumors were more similar than different at the molecular level, with only CRYBB2P1, a pseudogene, differentially expressed between populations. Among demographic characteristics, AAW consumed significantly lower amounts of caffeine and alcohol, were less likely to breastfeed and more likely to be obese.

**Conclusions:**

These data suggest that TNBC in AAW is not a unique disease compared to TNBC in CW. Rather, higher frequency of TNBC in AAW may, in part, be attributable to the effects of lifestyle choices. Because these risk factors are modifiable, they provide new opportunities for the development of risk reduction strategies that may decrease mortality by preventing the development of TNBC in AAW.

## Background

Although the majority of data generated from breast cancer research has come from studies using Caucasian women (CW) as subjects, it is becoming increasingly clear that the incidence, mortality, and length of survival after treatment for breast cancer vary greatly among different ethnic groups. Although overall incidence of breast cancer in the United States is higher for CW (125.4/100,000) than for African American women (AAW) (116.4/100,000) [1], breast cancer incidence is higher in young AAW compared to CW such that 30-40% of AAW with breast cancer are under age 50 when diagnosed compared to just 20% of CW [2]. In addition, the five-year survival rate for AAW (77%) is significantly lower than for CW (90%) [3] across all ages and tumor stages and subtypes, and the age-adjusted mortality rate for AAW (32.4/100,000) is the highest rate for any ethnic group studied [1].

Triple negative breast cancer (TNBC) is defined as tumors that do not express the estrogen or progesterone receptors or HER2. TNBC is an aggressive tumor phenotype, characterized by diagnosis at a younger age, high-tumor grade, larger mean tumor size, and higher rates of mortality compared to other tumor subtypes [4]. Several clinical trials are underway testing targeted agents, such as PARP, angiogenesis and EGFR inhibitors; however, to date cytotoxic therapy remains the standard treatment for patients with TNBC. TNBC is diagnosed significantly more frequently in premenopausal AAW (39%) compared to either postmenopausal AAW (14%) or in non-African Americans of any age (16%) [5]. This higher prevalence in young AAW coupled with higher mortality rates and lack of available targeted treatments provides an explanation, at least in part, for the less favorable outcomes of AAW with breast cancer [6].

A number of epidemiological risk factors have been associated with TNBC including reproductive factors such as younger ages at menarche and at first full-term pregnancy (FFTP), higher parity, and shorter (or lack of) duration of breastfeeding, as well as anthropometric factors such as higher body mass index (BMI) and waist-to-hip ratio [7]. In addition, gene expression differences have been detected in primary breast tumors between AAW and CW [8,9], although these studies were not limited to TNBC but included a range of tumor subtypes. Identification of both epidemiological and molecular factors that differ between AAW and CW with TNBC is critical to developing more effective risk reduction strategies as well as treatment options for AAW. To this end, differences in both a range of epidemiological factors including obesity, estrogen exposure, breastfeeding, diet and physical activity, and co-morbidities, as well as gene expression profiles were evaluated between AAW and CW with TNBC.

## Methods

### Patient enrollment and consent

For inclusion in the Clinical Breast Care Project (CBCP), all patients must have met the following criteria: 1) adult over the age of 18 years, 2) mentally competent and willing to provide informed consent, and 3) presenting to the breast centers with evidence of possible breast disease. Tissue and blood samples were collected with approval from the Walter Reed National Military Medical Center (WRNMMC) Human Use Committee and Institutional Review Board. All subjects enrolled in the CBCP voluntarily agreed to participate and were provided with layered consent forms that included permission to gather samples of breast and metastatic tissues and blood for use in future studies, and described the primary research uses of the samples.

### Data and specimen collection

Once informed consent was granted, nurse researchers interviewed enrollees in person to collect over 500 fields of demographic data. Completed questionnaires passed through quality assurance and the data was entered in a manual dual-data entry fashion into the Scierra CLWS database (Cimarron Software, Salt Lake City, UT). In addition to questionnaire information, tissue was collected from patients as previously described [10]. Diagnosis of every specimen was performed by a breast pathologist from hematoxylin and eosin (H&E) stained slides; staging was performed using guidelines defined by the AJCC Cancer Staging Manual seventh edition [11] and grade assigned using the Nottingham Histologic Score [12,13]. ER and PR status were determined by IHC analysis at a clinical laboratory (MDR Global, Windber, PA) and the percent stained cells were recorded. A cut-off of ≥1% was used to determine ER and PR positivity [14]. For HER2 status, IHC analysis was performed in the same clinical laboratory as ER and PR status (MDR Global, Windber, PA); cases with HER2 scores = 2+ were further evaluated by fluorescence in situ hybridization using the PathVysion® HER-2 DNA Probe kit (Abbott Laboratories, Abbott Park, IL) using HER2/CEP17 >2.2 to define positivity.

### Data generation and analysis

The CBCP database was queried to identify all female African American and Caucasian patients with TNBC diagnosed between 2001 and 2011 (n = 160). Demographic data collected at the time of enrollment, including reproductive and health history, and lifestyle choices, such as tobacco and alcohol use, exercise frequency, and fat intake were analyzed using chi-square analysis and Student’s t-tests. Survival analysis was performed using JMP 10 statistical software. Kaplan-Meier (product-limit) survival estimates were calculated for AAW, CW and both groups combined. All alive with disease (AWD), no evidence of disease (NED) and death from other causes (DOC) statuses were censored. A Log-Rank test was performed to test homogeneity of the survival estimates across AAW and CW. A *P-*value of 0.05 was used to determine significance.

To generate gene expression data, patients with available frozen tumor specimens were identified. H&E stained slides were examined by the pathologist and tumor areas marked for laser microdissection. Tumor samples were laser microdissected and gene expression data generated using HG U133A 2.0 arrays (Affymetrix, Santa Clara, CA) as previously described [8]. Microarray data was imported into Partek® Genomics Suite™ 6.5 (Partek, Inc, St. Louis, MO) as CEL files using default parameters. Raw data was pre-processed, including background correction, normalization and summarization using robust multi-array average (RMA) analysis and expression data log2 transformed. Differential expression analysis for the tumor specimens was performed using ANOVA with a False-Discovery Rate (FDR) <0.05, 2-fold change defining differential expression.

## Results

### Demographic and epidemiological characteristics of AAW and CW with TNBC

Of the 1,064 AAW and CW diagnosed with invasive breast cancer, 15% (n = 160) had TNBC. The frequency of TNBC was significantly higher (*P <* 0.001) in AAW (28%, 62/220) compared to CW (12%, 98/844). The average age at diagnosis was 52 years and did not differ significantly between AAW (50.9 years) and CW (53.1 years). The frequency of TNBC was higher in pre-menopausal (diagnosed <50 years) AAW (53%) compared to CW (42%), although this difference did not reach the level of significance.

When reproductive factors were evaluated, ages at menarche, first oral contraceptive use, and FFTP did not differ significantly between AAW (13.0, 20.4 and 23.1 years) and CW (12.8, 21.1 and 24 years), nor did length of contraceptive use or number of live births (73 months and 2.3 children in AAW; 75 months and 2.1 children in CW). Use of oral contraceptives and hormone receptor therapy (HRT), type of HRT, and parity did not differ significantly (Table 1). In contrast, there was a significantly lower frequency of parous AAW that ever breastfed compared to CW, although in those who did, length of breastfeeding did not differ significantly (10.4 and 10.5 months, respectively).

**Table 1:** Demographic and epidemiological characteristics of AAW and CW with TNBC

	**AAW (n = 62)**	**CW (n = 98)**	***P-*** **value**
Age at diagnosis			0.215
<40 years	0.15	0.07	
40–49 years	0.38	0.35	
≥50 years	0.47	0.58	
Oral contraceptive use			0.555
Yes	0.67	0.71	
No	0.33	0.29	
Parous			0.605
Yes	0.81	0.85	
No	0.19	0.16	
HRT use^a^			0.182
Yes	0.33	0.47	
No	0.67	0.53	
Type HRT used			0.971
Estrogen	0.31	0.30	
Estrogen and progesterone	0.54	0.52	
Unknown	0.15	0.18	
**Breastfeed**^**b**^			**0.001**
**Yes**	**0.33**	**0.63**	
**No**	**0.67**	**0.37**	
**BMI**			**0.024**
**<18.5**	**0.02**	**0.00**	
**18.5–24.9**	**0.27**	**0.23**	
**25–29.9**	**0.22**	**0.45**	
**30+**	**0.49**	**0.32**	
Fat intake^c^			0.731
	0.24	0.22	
	0.76	0.78	
Exercise			0.138
≤150 minute	0.80	0.69	
≥150 minutes	0.20	0.31	
Smoking			0.757
Never	0.63	0.57	
Past smoker	0.26	0.30	
Current smoker	0.11	0.13	
**Caffeine intake**			**<0.001**
**Safe/moderate (<500 mg/day)**	**0.60**	**0.30**	
**High/extremely high (≥500 mg/day)**	**0.40**	**0.70**	
**Alcohol consumption**			**0.029**
**none**	**0.46**	**0.27**	
**<1 drink/day**	**0.53**	**0.64**	
**1 drink/day**	**0.01**	**0.09**	
Education			0.794
College degree or higher	0.50	0.48	
Less than college degree	0.50	0.52	
Marital status			0.671
Married	0.64	0.68	
Not married	0.36	0.32	
Cardiovascular disease			0.623
Yes	0.03	0.02	
No	0.97	0.98	
Diabetes			0.080
Yes	0.16	0.07	
No	0.84	0.93	
Hypertension			0.147
Yes	0.40	0.29	
No	0.60	0.71	

^a^Evaluated in post-menopausal women only.

^b^Evaluated in parous women only.

^c^Assessed using the Northwest LRC Fat Intake Score.

Significant differences noted in bold.

Anthropometrically, AAW were significantly more likely to be obese. Fat intake [15], compliance with the recommended 150 minutes of exercise/week [16], and smoking histories did not differ significantly between populations. Caffeine intake was significantly lower in AAW (average 535 mg/day) compared to CW (average 1105 mg/day) and AAW were less likely to consume alcohol.

Education levels, marital status and presence of co-morbid conditions did not differ significantly between AAW and CW. Cardiovascular disease was not common in either population. Diabetes and hypertension were more common in AAW, although neither reached the level of significance.

### Pathological differences between TNBC tumors from AAW and CW

**Figure 1:**
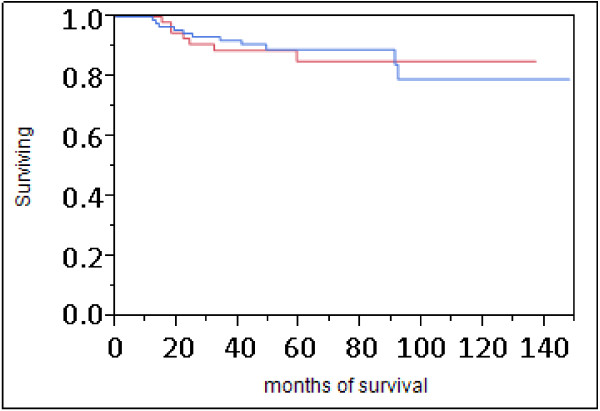
**Survival analysis of AAW and CW with TNBC.** Red line = AAW, blue line = CW. Statistical analysis by both log-rank (P = 0.9469) and Wilcoxen (P = 0.7273) testing failed to detect significant differences in survival between populations.

Tumors from AAW and CW did not differ significantly for stage, lymph node or Ki67 status (Table 2). Tumors were more likely to be of higher-grade and T2 tumor size, although these differences did not reach the level of significance. Twelve percent of patients in both populations died of disease and time between diagnosis and death did not differ significantly between AAW and CW. The average length of disease-free survival was 62.4 months in AAW and 61.3 months in CW. Overall survival did not differ significantly between populations (Figure 1).

**Table 2:** Pathological characteristics of AAW and CW with TNBC

	**AAW (n = 62)**	**CW (n = 98)**	***P-*** **value**
Stage			0.255
I	0.33	0.46	
II	0.48	0.36	
III	0.12	0.15	
IV	0.07	0.03	
Grade			0.160
Well-differentiated	0.02	0.03	
Moderately-differentiated	0.05	0.15	
Poorly-differentiated	0.93	0.82	
Size			0.072
T1	0.41	0.56	
T2	0.52	0.34	
T3	0.07	0.10	
Lymph node status			0.856
Positive	0.73	0.72	
Negative	0.27	0.28	
Ki-67^a^			0.889
Positive	0.16	0.17	
Negative	0.84	0.83	

^a^Tumors with Ki67 < 14% were considered negative, those ≥14% positive, as described by Cheang et al. [17].

### Gene expression profiling

**Figure 2:**
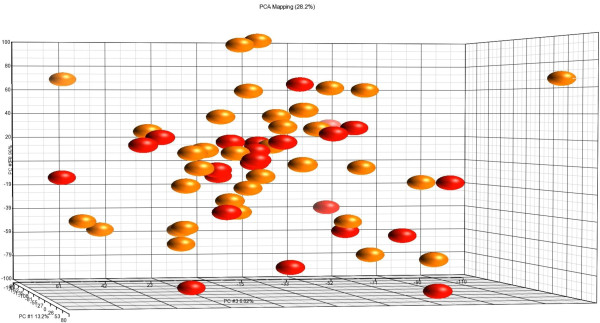
**Principal component analysis of TNBC from AAW (n = 23) and CW (n = 34).** Orange spheres = CW tumors, red spheres = AAW tumors.

**Figure 3:**
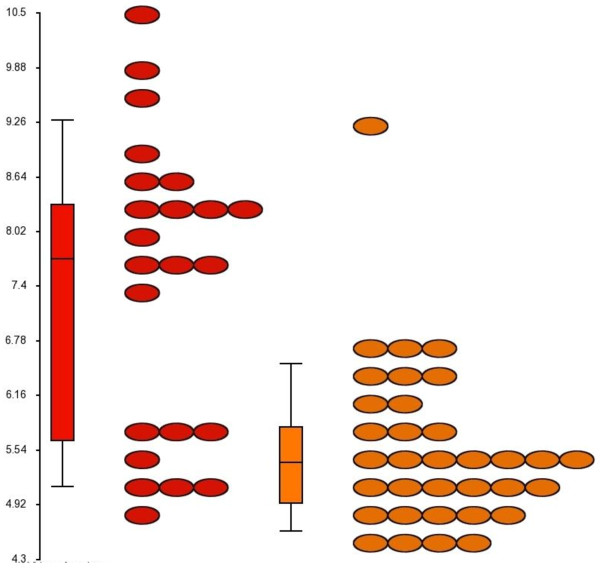
**Gene expression of probe 206777_s_at representing *CRYBB2P1.*** Red ovals = expression in AAW, orange ovals = expression in CW. Expression levels were 3.9-fold higher in AAW compared to CW, with 8/22 AAW having expression levels similar to CW.

Gene expression data was generated from 57 poorly-differentiated TNBC (23 AAW and 34 CW). Average age at diagnosis (51.3 and 53.3 years in AAW and CW, respectively) did not differ significantly between populations. Principal component analysis (PCA) failed to detect significant gene expression differences between populations (Figure 2). Only the probe for crystallin, beta B2 pseudogene 1 (*CRYBB2P1)* [GenBank: NR_033734], a pseudogene, was differentially expressed between populations with 3.9-fold higher expression in tumors from AAW (Figure 3). Hierarchical clustering revealed two clusters: the low CRYBB2P1 expression group included 33/34 CW and 8/23 AAW tumors and the high CRYBB2P1 expression group included 15/23 AAW and one CW tumor, resulting in a classification accuracy of 65% in AAW and 97% in CW.

## Discussion

To decrease survival disparities between AAW and CW with breast cancer, the source of outcome differences must be identified. Higher mortality rates have been detected for AAW in both the general population and the military when breast cancer was considered as a single disease [3,18], however, breast cancer is heterogeneous, with an array of phenotypic and molecular differences. Given the higher frequency of TNBC in AAW, higher mortality rates in AAW compared to CW with TNBC may explain outcome disparities between populations.

Data generated here do not support TNBC as a more aggressive disease in AAW. Mortality rates and length of disease-free survival did not differ significantly between populations. These results are supported by data from the Carolina Breast Cancer Study (CBCS) that demonstrated that while AAW had overall higher breast cancer mortality rates, when only patients with TNBC were considered, mortality rates did not differ significantly [19]. In addition, a recent study conducted at a single institution with similar treatment and follow-up between populations also failed to find differences in disease-free or overall survival between AAW and CW with TNBC [20]. Together, these data do not support TNBC as a clinically more aggressive tumor type in AAW compared to CW.

In conjunction with the inability to detect outcome differences between groups, TNBC tumors from AAW and CW were molecularly similar, with PCA failing to separate gene expression patterns by population. One gene, *CRYBB2P1*, was expressed at significantly higher levels in tumors from AAW compared to CW. *CRYBB2P1* has significant sequence similarity to *crystallin, beta B2*, a member of the crystallin gene family that encodes the major structural components of the vertebrate eye lens, however, CRYBB2P1 has been designated a pseudogene, and to date, the possible function of *CRYBB2P1* transcripts are unknown [21]. Higher expression of the probe for *CRYBB2P1* has been detected in a number of tissues from African Americans, including breast (of mixed subtypes), prostate and colorectal tumors, disease-free breast and prostate tissues [8,9,22,23] as well as blood endothelial cells [24]. Given the differential expression of this pseudogene in a variety of tissues, both malignant and non-malignant, additional studies must be performed to determine whether *CRYBB2P1* plays a causative role in tumorigenesis or reflects population stratification.

Although outcome disparities were not detected in this population, diagnosis of TNBC was significantly higher in AAW (28%) compared to CW (12%). Thus, identification of risk factors, both modifiable and non-modifiable, leading to the higher frequency of TNBC in AAW may reduce survival disparities by preventing the development of TNBC. For example, a SNP on chromosome 5p15 near the TERT locus was associated with TNBC in a mixed population of patients of African and European ancestries [25]; data from the Black Women’s Health Study (BWHS) confirmed this association and found that SNP rs8170 in the BABAM1 gene, was associated with increased risk of TNBC in an African American population [26]. A higher prevalence of the causative allele from these SNPs in women of African ancestry may explain the higher incidence of TNBC in AAW.

Modifiable risk factors that differed between populations in our study include caffeine and alcohol consumption, obesity and breastfeeding. In a study evaluating coffee and black tea consumption, a protective effect for coffee was found in pre-menopausal women, although this study was comprised of 98% Caucasian women [27]. In contrast, results from the BWHS failed to find an association between caffeine consumption and breast cancer risk, either overall or by menopausal or hormone receptor status [28]. Evaluation of alcohol consumption found a decreased risk of TNBC in alcohol consumers compared to non-drinkers and a significantly lower risk in those who consumed ≥7 drinks/week [29]. Thus, the possible protective advantages conferred by caffeine and alcohol consumption may not be realized by AAW, although more research is needed to definitively determine the benefits of caffeine and alcohol use in patients with TNBC.

A number of studies have evaluated the role of obesity on development of TNBC with mixed results. A pooled analysis of data from the Breast Cancer Association Consortium, which is comprised of 92% patients of European ancestry, did not detect an association between obesity and TNBC in case–control analysis of young women, although case-case analysis did find an association between obesity and TNBC in young women [30]. In contrast, associations between obesity and TNBC have been reported for patients not using hormone replacement therapy [31], and an elevated waist-hip ratio was associated with increased risk of basal-like breast cancers [32]. A recent meta-analysis found a significant association between obesity and TNBC in both case-case and case–control analyses, especially in pre-menopausal women [33]. With nearly half of our African American TNBC population having a BMI ≥30, this high incidence of obesity may contribute to the higher frequency of TNBC in AAW.

Breastfeeding, or lack thereof, has also been associated with increased risk of developing TNBC. Case-case analysis found that patients in the CBCS with TNBC breastfed for shorter durations than those with luminal A tumors, and case-controls analysis found an inverse relationship between breastfeeding and risk of TNBC [32]. A number of other studies have found an inverse association between breastfeeding and TNBC [34-37]. In our study, although the cumulative number of months spent breastfeeding did not differ significantly between parous AAW and CW, only 33% of parous AAW with TNBC ever breastfed, compared to 63% of CW. In contrast, significantly different rates of breastfeeding were not detected in 115 AAW and 596 CW with ER+/HER2- tumors enrolled in the CBCP, thus failure to breastfeed in parous women may be a risk factor specifically for the development of TNBC.

Limitations of this study include possible selection bias and provision of equal-access health-care. Despite having no protocols to specifically recruit any ethnic group into the program, the CBCP has been effective in enrolling AAW, who encompass 16% of female patients with invasive breast cancer. Data regarding the number of patients who declined enrollment were not available, thus whether participation in the CBCP differs between AAW and CW could not be determined. Factors associated with refusal to participate in clinical trials include mistrust of the medical community, lack of compliance with research protocols, and increased co-morbidities [38], thus, patients who agreed to participate in the CBCP may be healthier, more educated, and more compliant with short- and long-term treatments than those who did not. In addition, patients in the CBCP were provided with standardized health-care through the Department of Defense, which included screening mammograms, clinical breast exam, breast surgical procedures and chemo- and radiation therapies, regardless of ability to pay. Our study and that from Washington University [20] failed to find survival differences between AAW and CW with TNBC who received similar clinical care, suggesting that TNBC is not inherently a different disease in AAW, but reflect disparities in access to quality health-care.

## Conclusions

Overall survival, pathological characteristics and global gene expression patterns did not differ significantly between AAW and CW with TNBC, suggesting that TNBC is not intrinsically different between populations. In contrast, the frequency of TNBC was significantly higher in AAW compared to CW; because TNBC is an aggressive disease with comparably unfavorable outcomes in both AAW and CW, increased prevalence of TNBC in pre-menopausal AAW may be contributing to survival disparities. Understanding the genetic and environmental risk factors associated with higher rates of TNBC may be critical in the design of risk reduction strategies to reduce the burden of TNBC in the African American population; for example, data from the CBCS suggests that up to 68% of basal-like breast cancer could be prevented in young AAW with the promotion of breastfeeding and reduction of abdominal adiposity [32]. Together, these results suggest that TNBC is not a different disease in AAW compared to CW and that survival disparities attributed to more frequent diagnosis of TNBC in AAW may be best addressed with the development of targeted therapies for treating TNBC across populations and development of new risk reduction strategies to decrease the incidence in TNBC AAW.

## Competing interests

The authors declare that they have no competing interests.

## Authors’ contributions

LAS generated the microarray data and reviewed the manuscript, JM validated expression levels of CRYBB2P1 and reviewed the manuscript, KM performed statistical analysis and reviewed the manuscript, CDS provided patient samples and clinical interpretation of the data, REE designed the study and wrote the manuscript. All authors read and approved the final manuscript.

## Pre-publication history

The pre-publication history for this paper can be accessed here:

http://www.biomedcentral.com/1471-2407/14/62/prepub
